# The higher-order molecular organization of p62/SQSTM1

**DOI:** 10.18632/oncotarget.4590

**Published:** 2015-06-23

**Authors:** Terje Johansen, Carsten Sachse

**Affiliations:** European Molecular Biology Laboratory (EMBL), Structural and Computational Biology Unit, Heidelberg, Germany

The multifunctional signaling adaptor and selective autophagy receptor p62/SQSTM1 is commonly found in dense light-microscopic loci of eukaryotic cells. Recently, Ciuffa et al. demonstrated that p62 is able to form organized polymers of helical symmetry once purified and reconstituted in the test tube [1]. In selective autophagy, p62 acts both as a substrate and as a receptor to bridge LC3 attached to the autophagosomal membrane with ubiquitinated cargo destined for degradation in the lysosome [2]. In signaling, p62 acts as an adaptor protein by interacting with protein kinases (atypical protein kinase C, MEKK3, MEK5, ERK1 and RIP) and ubiquitin ligases such as TRAF6 and the KEAP1-Cul3 complex [3]. Hence, p62 is acting in important signaling pathways involving NFκB, NRF2 (NFE2L2), mTOR, Wnt and others. Evidence is accumulating that the signaling and autophagy roles of p62 come together in the regulation of inflammation, innate immunity, oxidative stress, cell death and tumorigenesis. p62 is located in polyubiquitin-containing protein aggregates in cultured cells and in brain tissues in neurodegenerative diseases and in liver cancers. In these situations the degradative autophagy machinery is overwhelmed and unable to cope with removal of excessive protein deposits [2, 4]. Oligomerization of p62 via the PB1 domain is required for formation of p62 bodies in cells and degradation by selective autophagy, and for signaling via the NRF2-mediated oxidative stress response pathway [2].

**Figure 1:**
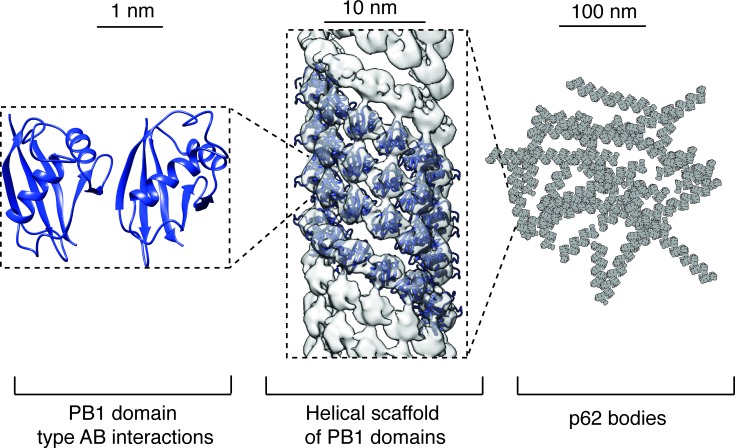
Structural hierarchy of p62/SQSTM1 organization The presented p62 structures span the size range from 1 nm to 100s of nm. Left. Dimer model from p62 rat homologue (PDB 2KKC). Center. PB1 domains assemble into polymers as helical strands that pack against each other to form helical assemblies (PDB 4UF8, EMD-2936). Right. Helical polymers are thought to make up the bulk of p62 bodies inside eukaryotic cells.

Structural studies on the protein had been hampered by the fact p62 formed aggregates mediated via the N-terminal PB1 domain. Thus, X-ray crystallographic and NMR structural information on the protein had only been obtained from isolated PB1 domains by preventing it to form large assemblies. While the central part of the protein containing the LC3 interacting region (LIR) is predicted to be disordered, the C-terminal part is composed of the folded UBA domain that recognizes ubiquitinated cargo and had only been structurally characterized in isolation. The recent structural analysis of Ciuffa et al. from a series of successively longer parts of p62 by electron cryo-microscopy (cryo-EM) revealed that the PB1 domain alone is sufficient to form helical polymers with significant flexibility and curvature (Figure 1) [1]. The cryo-EM maps resolved at 10.3 and 10.9 Å reveal a common helical strand of PB1 domains that is compatible with the canonical electrostatic interface consisting of acidic and basic patches elucidated by previous studies [5, 6].

When full-length p62 is assembled, it forms significantly shorter filaments or particles than PB1 domain polymers. Regions of predicted structural disorder and additional destabilization by the C-terminal part of the protein are likely to contribute to the observed heterogeneity of structures. In order to probe whether p62 helical polymers are capable to act as autophagy receptors, Ciuffa and co-workers tested whether they can bind LC3 and poly-ubiquitin. While LC3 binds to p62 helices, poly-ubiquitin binds and induces depolymerization of filaments. Similarly, over-expressed ubiquitin in RPE1 cells, lead to a reduction of p62 bodies *in vivo*. These findings open up ways to regulate the novel assembly state of p62. In this context, an electrostatic bridge C-terminal to the PB1 domain was identified that upon charge-reversal mutation suppresses the formation of long filaments, but favors the formation of oligomeric ring structures. Furthermore, it is possible to envision that post-translational modifications of residues in or close to the PB1 domain may act similarly as switches to modulate the assembly state of p62.

Helical polymers have a number of structural and biochemical properties that could make p62 filaments unique mediators in the context of autophagy and signaling. Large filamentous assemblies of p62 may provide the scaffold for the forming autophagosomal structure to encapsulate large cargos such as viruses, organelles and protein aggregates. Polymers possess multiple binding sites that increase the apparent avidity and selectivity ideally suited for the specific recognition of cellular binding partners. The entropic effect is particularly beneficial in autophagy, when protein ligands such as LC3 and poly-ubiquitin are also organized as an array of molecules on the membrane or in a linear chain.

Moreover, in signaling p62 acts as a molecular scaffold through PB1 domain interactions to create subcellular signaling loci (signalosomes) [7].

In order to better understand the implications of p62 filament assembly further studies are required to elucidate the mode of interactions with the binding partners. Structural studies on heterooligomeric or heteropolymeric assemblies will be essential to obtain mechanistic insights into the function of the observed p62 filaments. Although it has been established that p62 assembly is critical in autophagy and signaling function, disassembly will be an equally important regulatory mechanism to avoid excessive buildup of large p62 bodies in the cell. Insights into the mechanisms regulating assembly and disassembly of p62 filaments will have important implications for elucidating the roles played by p62 in vital cellular processes as well as in aberrant tumorigenesis.
